# Anticancer and Antioxidant Properties of *Vernonia amygdalina* Delile and *Citrus aurantifolia* (Christm.) Swingle Juice Extracts: An In Vitro Study

**DOI:** 10.1155/2024/9692656

**Published:** 2024-10-30

**Authors:** Eunice E. Ampem Danso, Eunice Dotse, Abigail Aning, Trudy Philips, Sherif Hamidu, Janet Ampofo

**Affiliations:** Department of Clinical Pathology, Noguchi Memorial Institute for Medical Research, College of Health Sciences, University of Ghana, P.O. Box LG 581, Legon, Ghana

**Keywords:** antiproliferative, cancer, cytotoxicity, extracts, inhibition, *Vernonia amygdalina*

## Abstract

**Introduction:** *Vernonia amygdalina* Delile (VAD), also known as bitter leaf, is widely utilized in traditional medicine for the treatment of various ailments, including cancer. The presence of bioactive compounds in VAD is believed to be responsible for its characteristic bitterness. In Ghana, it is a common practice to mitigate the bitterness of VAD by combining it with *Citrus aurantifolia* (Christm.) Swingle (lime) juice extracts, although this method lacks scientific evidence and documentation. Therefore, the antioxidant and anticancer activities of VAD and lime juice extracts (V5) and their combined effects were evaluated in vitro.

**Method:** The antioxidant activity and cytotoxic effects of VAD extracts were determined against Jurkat, MCF-7, HepG2, and PNT2 cells using the 2,2-diphenyl-1-picrylhydrazyl (DPPH) assay to quantify antioxidant activity and the 3-(4,5-dimethylthiazol-2-yl)-2,5-diphenyltetrazolium bromide (MTT) assay to assess cytotoxicity. The statistical analysis of the data was conducted using Microsoft Excel and GraphPad Prism 8.0. Linear regression was employed to determine the correlation between the concentration and the percentage of antioxidant activity, while *p* values were calculated using Student's *t*-test.

**Results:** The laboratory analysis focused on the extracts V1, V2, V3, V4, and V5. Briefly, V1 and V2 contained equal amounts of saponins and terpenoids. Among these, V2 exhibited the highest free radical scavenging activity, as indicated by an EC50 value of 2.14 ± 0.06 mg/mL. V2 also demonstrated cytotoxicity against the MCF-7, HepG2, Jurkat, and PNT2 cell lines. On the other hand, V3 and V4 did not show any cytotoxic effects across all tested cell lines. In contrast, V5 was toxic to HepG2 and MCF-7 cells but had no cytotoxic effect on Jurkat cell lines. V2 exhibited dose-dependent cytotoxicity (0–1000 *μ*g/mL), with the strongest inhibition observed against Jurkat cells (IC50 value = 96.341 *μ*g/mL) and a selective index of 3.567. The difference in activity between the extracts from different parts of the plant and the extract combined with lime juice was significant (*p* < 0.05), indicating a synergistic effect of the phytochemicals in both VAD and lime juice.

**Conclusion:** V2 and V5 demonstrated a remarkable antioxidant property, and they are effective in inhibiting cancer cell lines, respectively.

## 1. Introduction

Globally, cancer ranks as the second leading cause of death following cardiovascular disease, with the global mortality rate experiencing an increase from 12% in 1990 to 15% in 2013 [1]. Within Ghana, prevalent causes of cancer-related mortality encompass liver, prostate, lung, breast, ovarian, and leukemia cancers [2]. The advent of molecular targeted therapies, surgical techniques, radiation therapy, chemotherapy, and adjuvant therapy has substantially reduced the 5-year cancer mortality rate from 90% to 31% [3]. Nevertheless, existing cancer treatments exhibit certain limitations, including toxicity, multidrug resistance, and severe side effects, thereby underscoring the necessity for the development of novel and efficacious drugs [4].

Recent studies have provided evidence indicating that patients in certain hospitals in Ghana often present with advanced-stage conditions [5]. Subjective data gathered from these patients suggest that many initially choose traditional medicine as their first line of treatment, only turning to conventional healthcare when their conditions worsen [6]. Unfortunately, numerous alternative cancer treatments remain unapproved, and some are even considered hazardous due to their potential to interfere with conventional therapies and reduce the effectiveness of cancer drugs. Although evidence-based treatments in modern medicine have made significant progress in reducing cancer mortality rates, concerns persist regarding their effectiveness, cost, and adverse effects. As a result, many patients have sought complementary and alternative medicine (CAM) as an alternative treatment option [7]. While various medicinal plants have shown promise as anticancer agents, the scientific evidence supporting their efficacy is still limited, and numerous clinical trials suffer from inadequate study design [8].

Among these plants are *Vernonia amygdalina* Delile (VAD) and *Citrus aurantifolia* (Christm.) Swingle juice (lime juice, V5), which are utilized as therapeutic agents in traditional medicine either individually or in combination [9, 10]. The medicinal value of these plants lies in the presence of phytochemicals that elicit specific physiological actions in the human body. These phytochemicals include alkaloids, tannins, flavonoids, and phenolic compounds, which are known to possess antioxidant properties [11]. Antioxidant properties enhance the efficacy of anticancer agents by reducing oxidative stress in cancer cells [12]. The previous research collectively emphasizes the therapeutic potential of compounds exhibiting both antioxidant and anticancer properties, suggesting that such agents could offer a more effective strategy in the fight against cancer [13]. Another study discusses the mechanism by which certain compounds exert both antioxidant and anticancer activities, highlighting the importance of these dual-function agents in cancer therapy [14]. Every part of both plants contains complex active components such as anthraquinones, steroids, and cardiac glycosides, which possess pharmacological significance [15]. The leaves of these plants are green in color and have a distinct odor. VAD is commonly referred to as “bitter leaf” due to its bitter taste. This bitterness is attributed to bioactive constituents such as alkaloids, saponins, tannins, and glycosides. The bitterness can be mitigated either by boiling or soaking the leaves in multiple changes of water or by adding V5 [16]. In ethnobotanical medicine, the roots and leaves of VAD are used for their antibacterial, anticancer, antiparasitic, and antimalarial properties. They are also employed in the treatment of fever, hiccups, kidney problems, vomiting, intestinal illnesses, and stomach discomfort [17]. The roots and stems are utilized as chewing sticks in various West African countries, including Cameroon, Ghana, and Nigeria. The previous studies have demonstrated the hypoglycemic and hypolipidemic activities of VAD in experimental animals [18]. However, in Ghana, the combination of VAD and V5 extract is used for the treatment of a variety of ailments, including cancers, without scientific documentation. Therefore, the antioxidant and anticancer activities of VAD and lime juice extracts (V5), as well as their combined effects, were evaluated in vitro.

## 2. Materials and Methods

### 2.1. Plant Collection

Fresh leaves and roots of VAD and *C. aurantifolia* (Christm.) Swingle of high quality were collected from a backyard garden located in Afienya, Accra, where both plants were readily accessible.

### 2.2. Data Management and Statistical Analysis

The obtained data were subjected to regression analysis to determine the dose-response relationship between the extracts using Microsoft Excel and GraphPad Prism. Linear regression analysis was conducted to assess the correlation between the mean percent antioxidant activity and the anticancer activity of VAD extracts and various portions with lime juice. The results were presented as mean ± standard deviation (*n* = 3), and significance was determined using Student's *t*-test with *p* < 0.05 considered significant.

### 2.3. Ethical Consideration

Approval was obtained from the Ethical Review Board of Noguchi Memorial Institute for Medical Research (NMIMR) and Ghana Health Service (GHS) with the following approval numbers: 070/16-17 and GHS/RDD/ERC/admin/App/17/335, respectively.

### 2.4. Materials

#### 2.4.1. Cell Lines, Chemicals, and Reagents

The cell lines used were obtained from the Department of Clinical Pathology at NMIMR. Dimethyl sulfoxide (DMSO), 2,2-diphenyl-1-picrylhydrazyl (DPPH), 3-(4,5-dimethylthiazol-2-yl)-2,5-diphenyltetrazolium bromide (MTT) dye, trypan blue solution, absolute ethanol, HCl, methanol, isopropanol, phosphate buffer saline, 96-well plates, antibiotics (penicillin, streptomycin, and L-glutamine), fetal bovine serum (FBS), and culture media Dulbecco's modified eagle medium (DMEM) and RPMI were obtained from Sigma-Aldrich Company (St. Louis, Missouri, United States).

#### 2.4.2. Plant Material Identification

The roots and leaves of VAD were collected from the backyard garden in Afienya, Accra. A leaf specimen was used for taxonomic identification by a botanist at the Botany Department of the University of Ghana, Legon. A voucher specimen (EVAD-02) was deposited at the herbarium.

#### 2.4.3. Extraction of Plant Materials

The roots and leaves of VAD were separated and divided into two batches, each weighing 50 g. The first batch of plant materials was blended and extracted with 500 mL of distilled water through cold maceration for 72 h. Similarly, the second batch of roots and leaves were extracted with 495 mL of distilled water and 5 mL of lime juice for 5 min. After extraction, the resulting extracts were filtered, frozen, and freeze-dried. To test the solubility, 20 mg of the powdered extract was dissolved in 0.5 mL of absolute DMSO, ethanol, and methanol for 30 min. The highest yield was obtained with DMSO.

#### 2.4.4. Cell Culture and Treatment

Liver cancer cells (HepG2), leukemia cells (Jukart), breast cancer cells (MCF-7), and normal prostate cancer (PNT2) cell lines were obtained from the Department of Clinical Pathology, Noguchi Memorial Institute for Medical Research. MCF-7 and HepG2 cells were cultured in DMEM, while Chang and Jurkat cells were cultured in RPMI. Both media were supplemented with 10% FBS and 1% penicillin/streptomycin/L-glutamine. The cells were maintained in a humidified incubator with 5% CO_2_ concentration at 37°C and subcultured when they reached approximately 90% confluence.

#### 2.4.5. In Vitro Cytotoxicity Assay

The cytotoxicity of the DMSO extracts from the selected plant samples was evaluated against leukemia, breast cancer, liver cancer, and normal prostate cell lines. The modified MTT assay was performed to assess the cytotoxicity. RPMI and DMEM culture media were supplemented with 10% FBS containing penicillin, streptomycin, and L-glutamine, and the cells were cultured at 37°C in a humidified 5% CO_2_ atmosphere. The MTT assay was used to determine the cytotoxicity of VAD and *C. aurantifolia* extracts on both cancer and normal cell lines [19]. Cells were seeded into 96-well plates at a concentration of 1 × 10^5^ cells/well, treated with different concentrations of the plant extracts (0–1000 *μ*g/mL), and incubated for 72 h. A color control plate was set up for each extract, including the positive control, curcumin. After 4 h of incubation, the MTT solution (0.5 mg/mL in PBS) was added to each well on the plate. The reaction was stopped with acidified isopropanol solution, and the plate was left to incubate in darkness overnight at room temperature (26°C) before measuring the absorbance at 570 nm using a microplate reader (Tecan Infinite M200 Pro, Austria). The percentage cell viability was calculated using the following formula:

 $$\begin{array}{c} \%\text{cell viability }\frac{\text{mean absorbance }\left(\text{treated cells}-\text{blank}\right)}{\left(\text{untreated cells}-\text{blank}\right)}\times 100 \end{array}$$

The concentration that caused 50% inhibition of the various cell lines (IC50 values) was determined by plotting the percent cell viability on the *y*-axis against the extract concentrations on the *x*-axis.

#### 2.4.6. In Vitro Free Radical Scavenging Activity—DPPH Assay

The antioxidant activities of VAD leaf, root, and lime juice extracts were evaluated using the DPPH free radical scavenging assay [20]. Equal volumes (100 *μ*L) of each extract at various concentrations (0 to 10 mg/mL) were mixed with a methanolic solution of 0.5-mM DPPH. The mixture was incubated at room temperature for 20 min, after which the absorbance was measured at 517 nm using a Tecan Infinite M200 PRO plate reader (Austria). The IC50 value for each extract was calculated using the following formula:

 $$\begin{array}{c} \%\text{antioxidant activity}=\frac{\left(\text{A}0-\text{A}1\right)}{\text{A}0}\times 100 \end{array}$$

where A0 is the absorbance of the negative control (methanol/DMSO) and A1 is the absorbance of the test sample with DPPH. Butylated hydroxytoluene (BHT) was used as the standard control. Triplicate experiments were performed. The EC50 value, which is the concentration of the extracts that can cause 50% free radical scavenging activity, was determined.

#### 2.4.7. Phytochemical Analysis

Phytochemical analysis of each hydroethanolic plant extract was qualitatively done as described by Matos et al. [21] with minor modifications to determine the class of secondary metabolites present. These metabolites include saponins, terpenoids, tannins, phenols, alkaloids, and flavonoids. The presence or absence of each phytochemical was assessed using various tests: foam test for saponins, Salkowski's test for terpenoids, ferric chloride test for tannins, Mayer's test for alkaloids, and alkaline reagent test for flavonoids. The results were recorded as positive (+) for the presence and negative (−) for the absence of the respective phytochemicals.

#### 2.4.8. High-Performance Liquid Chromatography (HPLC) Fingerprint of VAD Extracts

HPLC fingerprint analysis of VAD was conducted as previously described by Elekofehinti et al. [22], with slight modifications. An Agilent 1100 system (Santa Clara, California, United States) equipped with a quaternary pump, autosampler, diode array detector (DAD), and HP ChemStation Software was used for the analysis. Chromatographic separation was performed on a Tskgel ODS C18 analytical column (250 × 4.6 mm i.d., 5 *μ*m particle size) maintained at 40°C. The mobile phase consisted of water with 0.1% phosphoric acid (A) and acetonitrile (B), with a flow rate of 0.7 mL/min.

The gradient program was as follows: 5%–10% B from 0 to 5 min, 10%–20% B from 5 to 10 min, 20%–50% B from 10 to 15 min, 50%–80% B from 15 to 25 min, 80% B from 25 to 30 min, 80%–5% B from 30 to 40 min, and 5% B from 40 to 45 min. The injection volume was 10 *μ*L. Samples were prepared at a concentration of 1 mg/mL in 50% methanol, then vortexed, sonicated for 10 min, and centrifuged at 12,000 rpm for 10 min. The wavelength was monitored at 254 nm.

## 3. Results

### 3.1. Antiproliferative Activity of VAD Extracts and Curcumin

The VA extracts demonstrated dose-dependent cytotoxicity against Jurkat, HepG2, MCF, and Chang cell lines. Notably, the combination of VAD leaf extract and lime juice was effective across all tested cell lines, showing the strongest inhibition against Jurkat (IC50 = 96.341 *μ*g/mL) and Chang (IC50 = 90.97 * μ*g/mL). Curcumin served as the standard reference compound.

### 3.2. Regression Analysis of DPPH Scavenging Activity and MTT Cell Viability

The relationship between the DPPH scavenging activity of the extracts and their antiproliferative effects on cancer cell lines was evaluated using linear regression analysis. The Pearson correlation coefficients were BHT: 0.89, V1: 0.98, V2: 0.91, V3: 0.99, V4: 0.96, and V5: 0.81, all with *p* values < 0.05.

As shown in Table 1, V2 exhibited cytotoxicity across all cell lines, with the strongest inhibition observed against Jurkat. In contrast, V5 showed cytotoxicity specifically against the HepG2 and MCF-7 cell lines.

## 4. Discussion

**Figure 1:**
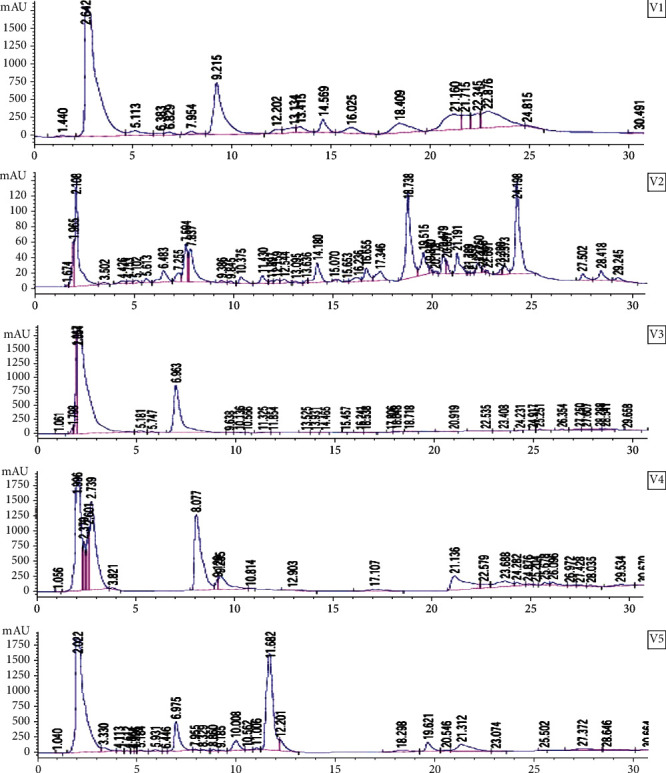
HPLC fingerprints of VAD extracts, lime juice extracts, and their combinations. This figure shows HPLC fingerprints of extracts (V1, V2, V3, V4, and V5) representing the phytochemical content of VAD extracts, lime juice extracts, and their combinations. The phytochemical content in each extract is ranked in descending order as follows: V2 > V3 > V5 > V4 > V1. V2 exhibited the highest phytochemical content, while V1 had the lowest.

A preliminary phytochemical screening was conducted on the 70% ethanolic extracts obtained from VAD leaves, roots, and lime juice, with sample labels V1, V2, V3, V4, and V5. The analysis revealed the presence of various secondary metabolites, including saponins, terpenoids, tannins, alkaloids, flavonoids, and phenols. Notably, V2 and V3 exhibited higher levels of saponins, while terpenoids were predominantly found in V3, V4, and V5. Tannins were only detected in V1 and V3, whereas flavonoids, alkaloids, and phenols were present in all samples. Interestingly, V1 displayed higher levels of alkaloids, and the phenol content increased in V1, V2, and V4. Saponins and terpenoids were identified as the most abundant secondary metabolites, followed by phenols, alkaloids, and flavonoids in all extracts (Table 2). The previous studies have already reported the antioxidant properties and potential cancer risk–reducing effects of these phytoconstituents [23, 24]. The presence of these phytoconstituents was further verified through HPLC fingerprinting, wherein V2 exhibited the greatest number of peaks (44). V3 and V5 followed closely with 35 and 32 peaks, respectively, while V4 displayed 29 peaks. V1, in contrast, demonstrated the fewest peaks (Figure 1).

**Figure 2:**
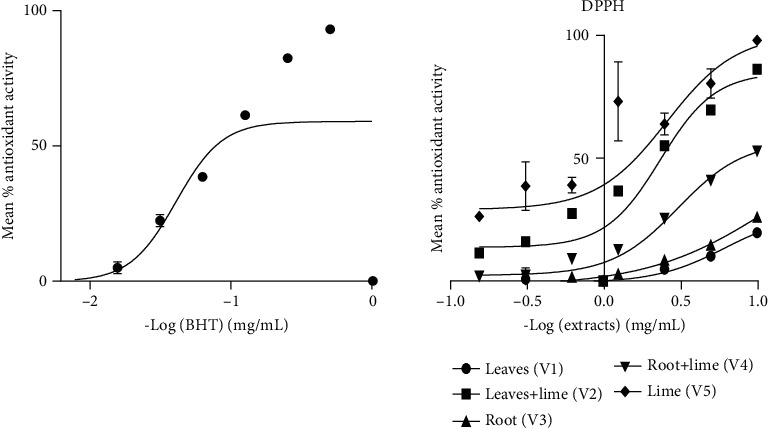
Antioxidant activity of BHT, VAD leaf extract, root extract, and mixtures of leaf and lime and root and lime extracts. Each point represents the mean of three determinations. This figure shows the EC50 values of BHT, V1, V2, V3, V4, and lime as follows: 0.093 ± 0.01, > 10, 2.14 ± 0.06, > 10, 8.63 ± 0.07, and 0.87 ± 0.16, respectively. The extract exhibits antioxidant and free radical scavenging properties. The extract possesses significant antioxidant and free radical scavenging properties (*p* < 0.05), with varying potency among the different samples in descending order of free radical scavenging activity: BHT > lime (V5) > V2 > V3, V1, and V4. The lower the EC50 value, the higher the free radical scavenging activity.

**Figure 3:**
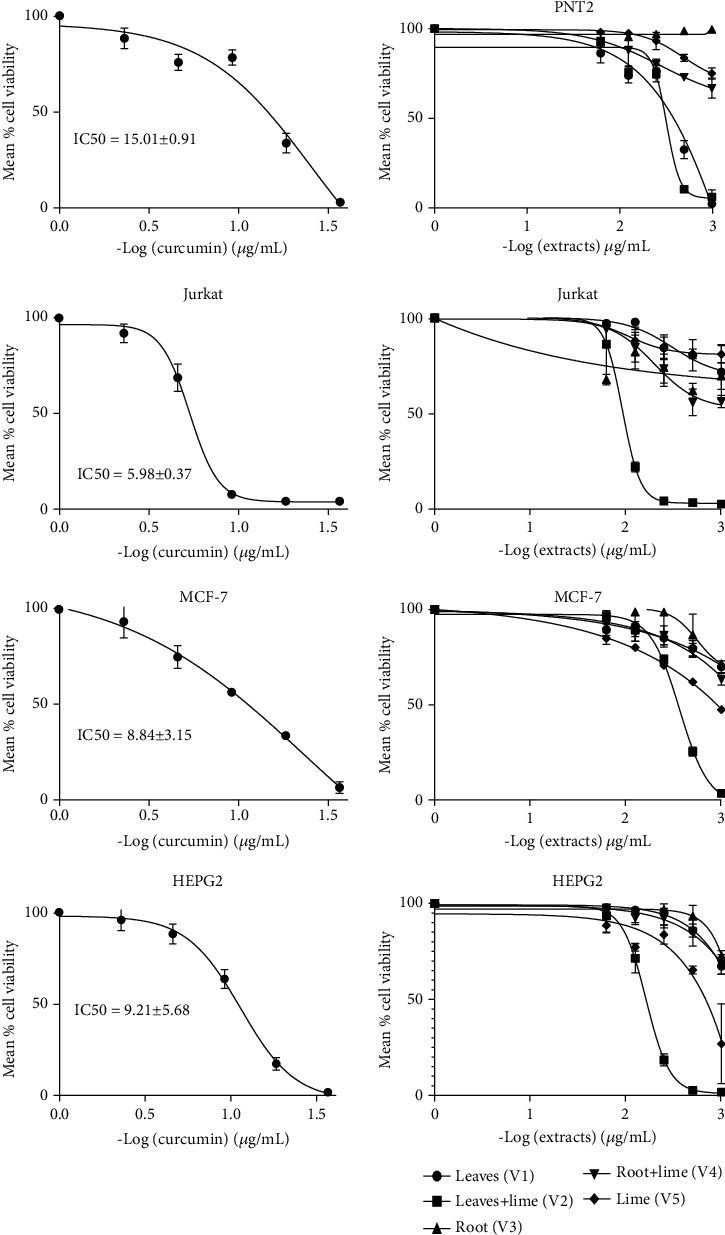
Antiproliferative activity of VAD extracts against Jurkat, MCF-7, HepG2, and PNT2 cell lines. This figure shows the antiproliferative activity of extracts against cell lines (Jurkat, MCF-7, HepG2, and PNT2). V1 (leaf extract) exhibited toxicity towards the normal cell line (PNT2). V2 (leaves + lime) and V5 (lime) displayed pronounced toxicity across all cell lines. V3 (root extract) and V4. Root + lime demonstrated reduced toxicity against cell lines, including the normal cell lines (PNT2).

The present study employed the DPPH and MTT assays (Figures 2 and 3) to evaluate the antioxidant and antiproliferative properties of the extracts [25]. The growth inhibitory effects of the extracts were assessed on four different cell lines: PNT2 (normal prostate cells), MCF-7 (breast cancer cells), Jurkat (leukemia cells), and HEPG2 (liver cancer cells). Among the extracts, V2 and V5 exhibited the highest free radical scavenging activity, which can be attributed to the presence of secondary metabolites in VAD and V5, as indicated in Table 2 and Figure 1. The previous research has highlighted the potential of phytochemicals, particularly phenolic compounds as effective antioxidants and free radical scavengers [26]. Furthermore, this study demonstrated the diverse range of biological activities exhibited by polyphenols, including their anticancer effects [4].

V2 exhibited dose-dependent inhibition across all cell lines tested (0–1000 *μ*g/mL), with the most significant inhibition observed in Jurkat cells (*p* < 0.05), resulting in a selectivity index greater than 2 (Table 1). This finding is consistent with previous studies that reported the susceptibility of Jurkat cells to medicinal plant extracts, such as *Sutherlandia frutescens* leaf extract, compared to MCF-7 and PC3 cancer cell lines [27]. Additionally, the combination of VA and V5 significantly enhanced the cytotoxic effect of V2 on PNT2 cells (*p* < 0.05). The previous studies have shown that V5 and VA extracts contain a substantial number of phytochemicals that exhibit significant antioxidant and anticancer properties [10]. The present findings are in line with the data provided in Tables 1 and 2, as well as Figures 1 and 2, underscoring the therapeutic potential and advantages of combining therapies to address the issue of antidrug resistance. Li et al. [28] have emphasized that combination therapy enhances the effectiveness of cancer treatment by simultaneously targeting multiple pathways involved in tumor growth and survival, thus delaying or preventing the emergence of drug resistance.

The results obtained from V2 indicate a potential synergistic effect between the phytochemicals present in V1 and V5, as evidenced by their augmented anticancer and antioxidant activities. Nevertheless, it is important to acknowledge that V2's cytotoxicity towards PNT2 cells raises concerns about its potential toxicity, a common challenge encountered in cancer therapies [1]. In the context of combination therapy, one compound may hinder the metabolic activation of another, resulting in an accumulation of toxicity [29]. Furthermore, V2 is rich in terpenoids and saponins, both known for their cytotoxic effects on cancer cells. The combination of these phytochemicals may disrupt the cytoskeleton, induce autophagic cell death, and reduce nitric oxide production [30]. These findings highlight the intricate interactions between different phytochemicals and their diverse effects on both cancerous and normal cells.

To enhance the observed cytotoxicity on cancer cells while minimizing toxicity to normal cells, further purification of the VAD leaf fractions could be beneficial. Additionally, V5 demonstrated cytotoxicity against the MCF-7 and HEPG2 cell lines, whereas compound V4 had no impact on any of the tested cell lines, including Jurkat cells (IC50 > 1000 *μ*g/mL).

Similarly, V3 demonstrated no toxicity towards Jurkat and HEPG2 cells (IC50 > 1000 * μ*g/mL), despite having higher levels of terpenoids and saponins. The absence of observed toxicity in V3 could be attributed to the antagonistic interaction between saponins and alkaloids, as reported in literature [31]. Interestingly, despite V1 containing similar levels of saponins and alkaloids, it demonstrated cytotoxic effects against PNT2 cells. Boța et al. [32] highlighted the potential for alkaloids and saponins to exert synergistic effects under certain conditions. However, their study also noted that the combined effect of these compounds on normal cell lines led to significant cytotoxicity.

The inhibitory effects of tannins on the proliferation of cancer cells are widely acknowledged; however, the specific outcomes depend on the composition and dosage of tannins used [33]. Although tannins were absent in both V2 and V4, their toxicity profile differs significantly. V2 exhibited toxicity towards normal cell lines. In contrast, the half maximal inhibitory effect of V4 was > 1000, indicating a lack of cytotoxic activity. This may explain the reduced anticancer activity observed in extracts V1, V3, and V4. Additionally, combining different drug molecules or plant constituents can potentially enhance the side effects of each component [34]. A strong positive Pearson's correlation was observed between the cytotoxic effects of extracts on cancer cell lines and their ability to scavenge free radicals, as determined through linear regression analysis. However, literature presents varying results regarding the role of antioxidants in the prevention and treatment of cancer [35]. The antioxidant activity of medicinal plant extracts proves beneficial in cancer therapy. Nevertheless, the cytotoxic effects observed in the MTT cell viability assay conducted in this study may stem from mechanisms beyond the antioxidant activity of VAD. These mechanisms may encompass the modulation of signalling pathways, alteration of cellular metabolism, or targeting specific processes within cancer cells.

The recent studies suggest that the biological context plays a crucial role in determining whether antioxidants hinder or facilitate tumorigenesis. This emphasizes the paradoxical impact of antioxidants on cancer development [36].

## 5. Conclusion

The study identified key secondary metabolites, including saponins, terpenoids, tannins, alkaloids, flavonoids, and phenols. Among these, saponins and terpenoids were found to be the most abundant. HPLC fingerprinting provided further support for these findings, demonstrating that V2 exhibited the highest number of peaks, indicative of a diverse phytochemical profile. Antioxidant and antiproliferative assays revealed that V2 and V5 possessed significant free radical scavenging activity, likely attributable to their rich phytochemical content. The mixture of V1 and V5 displayed a dose-dependent inhibition across various cancer cell lines, with notable efficacy against Jurkat cells, suggesting its potential as a therapeutic agent. Furthermore, the combination of V1 and V5 highlighted the potential synergistic benefits of employing multiple extracts in tandem. Fractionation and purification of V1 could potentially position V2 as a promising option for chemotherapy in the treatment of leukemia, breast cancer, and liver cancer. However, it is crucial for patients considering V2 as a cancer treatment option to exercise caution and prioritize safety.

## Figures and Tables

**Table 1:** Selectivity index (S.I.) of VAD extracts against cell lines.

**Samples**	**Cell line**						
	**PNT2**	**HEPG2**		**MCF-7**		**Jurkat**	
	**IC50**	**IC50**	**S.I.**	**IC50**	**S.I.**	**IC50**	**S.I.**
Curcumin	15.014	9.207	1.630	8.838	1.699	5.984	2.509
VI	401.009	⁣^∗∗∗^	⁣^∗∗∗^	⁣^∗∗∗^	⁣^∗∗∗^	⁣^∗∗∗^	⁣^∗∗∗^
V2	343.523	174.056	1.974	374.275	0.918	96.341	3.567
V3	> 1000	⁣^∗∗∗^	⁣^∗∗∗^	⁣^∗∗∗^	⁣^∗∗∗^	⁣^∗∗∗^	⁣^∗∗∗^
V4	> 1000	⁣^∗∗∗^	⁣^∗∗∗^	⁣^∗∗∗^	⁣^∗∗∗^	⁣^∗∗∗^	⁣^∗∗∗^
V5	> 1000	666.626	1.500	907.288	1.102	> 1000	

*Note:* This table illustrates the toxicity profile of V1, V2, V3, V4, and V5 against normal (PNT2) and cancer cell lines. V2 and V5 demonstrated strong inhibition across all cell lines, with an S.I.<2 against HEPG2 and MCF-7, comparable to curcumin. Notably, V2 exhibited the strongest inhibition against Jurkat, with an S.I.>2 (3.57), surpassing the S.I. (2.51) of curcumin against Jurkat.

⁣^∗∗∗^Half maximal inhibitory concentration > 10,000.

**Table 2:** Phytochemical analysis of extracts.

**Sample**	**Saponins**	**Terpenoids**	**Tannins**	**Flavonoids**	**Alkaloids**	**Phenolics**
V1	++	++	+	+	++	++
V2	+++	+++	−	+	+	++
V3	++++	+++	++	+	+	+
V4	+	+++	−	+	+	++
V5	++	++++	−	+	+	++

*Note:* This table presents the phytoconstituents of the extracts. VAD extracts (V1, V3), *C. aurantifolia* (V5), and their combinations (V2, V4) contain a range of phytochemicals. Extracts exhibited the highest levels of terpenoids, with saponins being the second most abundant. V1: leaf extracts; V2: leaf and lime juice extracts; V3: root extracts; V4: root and lime juice extracts; V5: lime juice extracts.

## Data Availability

The datasets used and/or analyzed during the current study are available from the corresponding author on a reasonable request.

## References

[1] Huang J., Zhang J., Sun C. (2024). Adjuvant role of *Salvia miltiorrhiza* bunge in cancer chemotherapy: a review of its bioactive components, health-promotion effect and mechanisms. *Journal of Ethnopharmacology*.

[2] Mensah K. B., Mensah A. B. B. (2020). Cancer control in Ghana: a narrative review in global context. *Heliyon*.

[3] Zafar A., Khan M. J., Abu J., Naeem A. (2024). Revolutionizing cancer care strategies: immunotherapy, gene therapy, and molecular targeted therapy. *Molecular Biology Reports*.

[4] Bédoui I., Nasr H. B., Ksouda K. (2024). Phytochemical composition, bioavailability and pharmacokinetics of Scorzonera undulata methanolic extracts: antioxidant, anticancer, and apoptotic effects on MCF7 cells. *Pharmacognosy Magazine*.

[5] Wiafe E., Mensah K. B., Appiah K. A. A., Oosthuizen F., Bangalee V. (2022). The direct cost incurred by patients and caregivers in diagnosing and managing prostate cancer in Ghana. *BMC Health Services Research*.

[6] Okyere Asante M. K. (2022). Classics and the politics of Africanization in Ghana. *Bulletin of the Institute of Classical Studies*.

[7] Singh S., Verma D., Goupale C. (2024). Exploring Medical Pluralism as a Multifaceted Approach to Healthcare. *Indian Journal of Integrative Medicine*.

[8] Bouyahya A., Bakrim S., Aboulaghras S. (2024). Bioactive compounds from nature: antioxidants targeting cellular transformation in response to epigenetic perturbations induced by oxidative stress. *Biomedicine and Pharmacotherapy*.

[9] Mohammed A., Amsalu B., Hailu M. (2024). Indigenous herbal medicine use and its associated factors among pregnant women attending antenatal care at public health facilities in Dire Dawa, Ethiopia: a cross-sectional study. *BMJ Open*.

[10] N’guessan B. B., Asiamah A. D., Arthur N. K. (2021). Ethanolic extract of *Nymphaea lotus* L. (Nymphaeaceae) leaves exhibits in vitro antioxidant, in vivo anti-inflammatory and cytotoxic activities on Jurkat and MCF-7 cancer cell lines. *BMC Complementary Medicine and Therapies*.

[11] Igwe O. U., Oru C. C., Otuokere I. E. (2024). Chemical and bioprotective studies of Xylopia aethiopica seed extract and molecular docking of doconexent and cryptopinone as the prominent compounds. *African Scientific Reports*.

[12] Kumar H., Kumar D., Kumar P. (2022). Synthesis, biological evaluation and in-silico ADME studies of novel series of thiazolidin-2,4-dione derivatives as antimicrobial, antioxidant and anticancer agents. *BMC Chemistry*.

[13] Zhang P., Wei W., Zhang X., Wen C., Ovatlarnporn C., Olatunji O. J. (2023). Corrigendum to “Antidiabetic and antioxidant activities of Mitragyna speciosa (kratom) leaf extract in type 2 diabetic rats” [Biomed. Pharmacother. 162 (2023) 114689]. *Biomedicine and Pharmacotherapy*.

[14] Rahmouni F., Hamdaoui L., Saoudi M., Badraoui R., Rebai T. (2024). Antioxidant and antiproliferative effects of *Teucrium polium* extract: computational and *in vivo* study in rats. *Toxicology Mechanisms and Methods*.

[15] Zia M., Parveen S., Shafiq N. (2024). Exploring *Citrus sinensis* phytochemicals as potential inhibitors for breast cancer genes BRCA1 and BRCA2 using pharmacophore modeling, molecular docking, MD simulations, and DFT analysis. *ACS Omega*.

[16] Dandi S. O., Abarike E. D., Ampofo-Yeboah A. (2022). Bitter leaf *Vernonia amygdalina* extract enhances growth, hematology, heat stress response, and resistance to *Aeromonas hydrophila* in Nile tilapia. *North American Journal of Aquaculture*.

[17] Sibeko L., Johns T., Hsiao B. S. (2023). Traditional perinatal plant knowledge in sub-Saharan Africa: comprehensive compilation and secondary analysis. *South African Journal of Botany*.

[18] Abonyi M. C., Ugwu T. E., Ezeude C. M. (2024). Prevalence and Determinants of complementary and alternative medicine use in subjects with hypertension in a tertiary centre in south east Nigeria. *Asian Journal of Medical Principles and Clinical Practice*.

[19] Ahmad F., Surapaneni K. M., Kamaraj B., Kamaraj B. (2024). Anti-proliferative activity and apoptotic induction of tannins extracted from *Quercus* infectoria on oral cancer KB cell lines. *Journal of Applied Pharmaceutical Science*.

[20] Cheleng N., Hanwar D. (2024). *DPPH free radical scavenging activity of lempuyang wangi leaves (Zingiber aromaticum. Val) extracts and fractions*.

[21] Matos P., Paranhos A., Oliveiros B., Cruz M. T., Batista M. T., Figueirinha A. (2024). Biological and phytochemical variation with pre- and post-harvest conditions for the *Acanthus mollis* L. leaf. *Industrial Crops and Products*.

[22] Elekofehinti O. O., Akintoye O. N., Akinjiyan M. O. (2024). Effect of *Carpolobia lutea* leaf extract on erectile dysfunction induced by paroxetine in rats. *Phytomedicine Plus*.

[23] Edo G. I., Samuel P. O., Jikah A. N. (2023). Biological and bioactive components of bitter leaf (*Vernonia amygdalina* leaf): insight on health and nutritional benefits. A review. *Food Chemistry Advances*.

[24] Ampem Danso E. E. (2018). *Anticancer And Antioxidant Properties of Vernonia Amygdalina*.

[25] Ampem Danso E. E. (2018). *Anticancer and Antioxidant Properties of Vernonia Amygdalina*.

[26] Muscolo A., Mariateresa O., Giulio T., Mariateresa R. (2024). Oxidative stress: the role of antioxidant phytochemicals in the prevention and treatment of diseases. *International Journal of Molecular Sciences*.

[27] Canga I., Vita P., Oliveira A. I., Castro M. Á., Pinho C. (2022). In vitro cytotoxic activity of African plants: a review. *Molecules*.

[28] Li J., Hu H., Lian K. (2024). CAR-NK cells in combination therapy against cancer: a potential paradigm. *Heliyon*.

[29] Abd El-Hafeez T., Shams M. Y., Elshaier Y. A. M. M., Farghaly H. M., Hassanien A. E. (2024). Harnessing machine learning to find synergistic combinations for FDA-approved cancer drugs. *Scientific Reports*.

[30] Khattak M., Khan T. A., Nazish M. (2024). Exploration of reducing and stabilizing phytoconstituents in *Arisaema dracontium* extract for the effective synthesis of silver nanoparticles and evaluation of their antibacterial and toxicological proprties. *Microbial Pathogenesis*.

[31] Gassim H. B. M., Hassan A. M., Abadi R. S. M., Mustafa Y. A. A. (2024). Phytochemical constituents and antioxidant activity of Ricinus communis Linn leaf and seeds extracts. *Scientiae Radices*.

[32] Boța M., Vlaia L., Jîjie A. R. (2024). Exploring synergistic interactions between natural compounds and conventional chemotherapeutic drugs in preclinical models of lung cancer. *Pharmaceuticals*.

[33] Hu Q., Wang S., Cheng R. (2024). Tannins in *Phyllanthus emblica* L. improves cisplatin efficacy in lung cancer cells by boosting endoplasmic reticulum stress to trigger immunogenic cell death. *Phytomedicine*.

[34] Wei Q., Li P., Yang T. (2024). The promise and challenges of combination therapies with antibody-drug conjugates in solid tumors. *Journal of Hematology and Oncology*.

[35] Haines D. D., Cowan F. M., Tosaki A. (2024). Evolving strategies for use of phytochemicals in prevention and long-term management of cardiovascular diseases (CVD). *International Journal of Molecular Sciences*.

[36] Noel K. I. (2024). A Reciprocal Relationship between Oxidative Stress, Antioxidants, and Cancer: a review. *Siriraj Medical Journal*.

